# Supplementary material for: StrIPETrack: a real-time, ROI-flexible tracking platform for high-throughput zebrafish behavior
Source: Biol Open. 2026 Apr 24;15(4):bio062503. doi: 10.1242/bio.062503 (PMC13148472; doi:10.1242/bio.062503)
Supplement: Supplementary information [file biolopen-15-062503-s1.pdf]

Contents

1 **Structure** 2

2 **Real Time Tracking** 2

3 **Tracking Videos** 2

4 **ROI Selector DearPyGUI** 2

5 **Y-Maze Analysis** 3

6 **96-well plate Analysis** 3

7 **Structural Similarity** 5

8 **Supplementary Figures** 6

1 Structure

To reduce complexity and increase modularity, the code has been divided into several Git submodules which can be used independently. Namely,

1. roi_selector_dearpygui - GUI for selecting desired ROIs
2. strsim_for_speed - structural similarity (computer vision method used)
3. y_maze_analysis - analysis of Y maze results & visualization

2 Real Time Tracking

For our tracking setup, we use a Grasshopper Gig-E camera. We access the camera using PySpin, a wrapper of Spinnaker. We provide both a GUI and a non-GUI interface for live-tracking, which are easily adapted to other cameras.

3 Tracking Videos

To track a folder of videos, simply open it using video-display-ex.py by inputting folder name into parameter '–folder'. This will open GUI, and you can save .cells files for each video in the folder. Next, run each video in the folder using str_sim_run.py. This will output a pre & post-processed csv containing position data [pre contains all candidates considered for zebrafish, post is the final position], as well as csvs containing frame & detected arm, and frame & detected turn (L/R).

4 ROI Selector DearPyGUI

GENERAL FUNCTIONALITIES

Delete: To delete any object, simply press 'delete' while hovering over the object.
Copy: 'CTRL+C' copies the object that is being hovered over - they are not offset, so if you press 'CTRL+C' and nothing appears to happen, try to drag the object.
Move All Objects: Both ROIs and Lines can be shifted using the WASD keys, and every ROI/Line will be affected. This behavior is segregated s.t. if you are in ROI mode, it will only affect ROIs, and if you are in Line mode, it will only affect lines. W == UP, A == RIGHT, S == DOWN, D == LEFT
Save: Opens a directory tool, saves all current ROIs or lines to given filename.

LINE MODE

Move Line: To move any line, hover the mouse tool over the center point (or either vertex), click, and drag.
Generate ROIs: Makes ROIs using lines as ROI bounds [see Fig.1]. After

Biology Open • Supplementary information

all ROIs are generated, all behaviors of ROIs still apply (i.e., you can still use WASD to move ROIs, 'del' to delete them, etc.)
Clear All ROIs: Deletes all current ROIs, returns to initial state.

ROI MODE
Move ROI: To move any ROI, simply hover the mouse tool over it, click, and drag - when you release the mouse tool, the ROI will be dropped in place.
Rotate ROI: To rotate any ROI, simply hover over any vertex of the ROI, click and drag.
Drag ROI points: To manipulate the size of the polygon itself (ex: if you made the ROI too small), click the 'Allow Point Drag' checkbox. You can now click and drag the vertices of any ROI to move it. To deselect this option, simply click the checkbox again.

What happens when I try to move an ROI out of bounds?
The ROI will be placed back within bounds.

Expanding or modifying:
To change the appearance of the GUI itself, modify either video_gui [if you are attempting to change the video's visualization] or gui [to modify the GUI itself]. The relationship between the ROI Interface and Line interface, is mediated by state_manager.

5 Y-Maze Analysis

To generate all figures listed in this paper, use paper_figures.ipynb or paper_figures.py.

Arm Analysis
Arm analysis takes in a csv with at minimum columns labeled 'pos_x', 'pos_y', 'frame', 'row' and 'col', and a .cells file, a pickle of every ROI. It outputs csvs containing arm data and tetragram data.

6 96-well plate Analysis

Parameters
-xhym - creates position based heatmaps
-e - same scheduled events used to schedule stimuli for run
-g - genotype ids, shown below
-s - sections file, shown below
-j - parameters for plotting
-r - cells file, generated by GUI
-m - path to experiment folder
-output_folder - output folder for data and graphs

-c - centroid file

-d - dpix file (LABVIEW only)
-t - timestamp file (LABVIEW only)

Python uses csv file containing positions, timestamps, and dpix, and inputs it here. LabVIEW splits those up into a postions file (.centroid), a dpix file (.motion) and a timestamp file (.timestamp).

Parameter Name	Python	LabVIEW	Details
-xyhm	x	x	
-e	x	x	
-c	x	x	csv vs .centroid
-d		x	.motion
-t		x	.timestamp
-g	x	x	
-s	x	x	standard sections file in Git
-j	x	x	PlotParameters example in Git
-r	x	x	file ending in '.cells' vs a rois_string
-m	x	x	

EXAMPLE PYTHON CALL:
python processmotiondata.py -python -xyhm -e /path/to/file/scheduled-events -c /path/to/file/pre-processed.csv -g /path/to/file/genotype_file -s /path/to/file/sectionsfile -j PlotParameters -r /path/to/file/zebrafish-tracker.cells -m /path/to/location/of/highspeedmovies/
EXAMPLE LABVIEW CALL:
python processmotiondata.py -xyhm -e /path/to/file/fulltestrun_final_01_27_2020 -c /path/to/file/'testlog.centroid1.Tue, Dec 23, 2025' -d /path/to/file/'testlog.motion1.Tue, Dec 23, 2025' -t /path/to/file/'testlog.timestamp1.Tue, Dec 23, 2025' -g /path/to/file/genotype_file -s /path/to/file/sectionsfile -j PlotParameters -r /path/to/file/rois_string -m /path/to/location/of/highspeedmovies/

genotyping
name_of_group:1,2,3,4 ...
other_name_of_group:10,11,12,13 ...

sections file : pulls out specific times for analysis
lightflash_day5dpflfday=1_9:10:00-1_9:26:00
ppi_day5dpfppi=1_9:37:00-1_15:00:00
habituation_day5dpfhab1pre=1_15:35:00-1_15:54:00
habituation_day5dpfhab1=1_16:03:00-1_16:05:00
...

Biology Open • Supplementary information

7 Structural Similarity

SSIM (assuming $\alpha = \beta = \gamma = 1$ and $C3 = \frac{C2}{2}$) can be implemented as follows:
$L =$ max data range (i.e. 255), $K1 = 0.01$, $K2 = 0.03$

$C1 = (K1 * L)^2$
$C2 = (K2 * L)^2$

$\mu_x = mean(im_1)$
$\mu_{xx} = mean((im_1)^2)$
$\mu_{xy} = mean(im_1 im_2)$
$\mu_y = mean(im_2)$
$\mu_{yy} = mean((im_2)^2)$

$\sigma_x = (cov)(\mu_{xx} - \mu_x^2)$
$\sigma_y = (cov)(\mu_{yy} - \mu_y^2)$
$\sigma_{xy} = (cov)(\mu_{xy} - \mu_y \mu_x)$

$$diff = \frac{(2\mu_x\mu_y + C1)(2\sigma_{xy} + C2)}{(\mu_x^2 + \mu_y^2 + C1)(\sigma_x + \sigma_y + C2)}$$

When using SSIM for tracking on a video, you can improve speed by comparing against a mode image or comparing the previous image taken to the current image taken. Based on the set of equations above, it's clear that μ_x, μ_{xx}, and σ_x are all derived from im1 alone, and μ_y, μ_{yy}, and σ_y are all derived from im2 alone.

Mode SSIM: Assuming im1 is a mode image that is not recalculated for every frame, μ_x, μ_{xx}, and σ_x will remain the same until mode is updated.

Prev SSIM: By the same trick - if you want to compare each previous frame to each current frame, taking im1 as the current image, μ_y, μ_{yy}, and σ_y will become μ_x, μ_{xx}, and σ_x.

8 Supplementary Figures

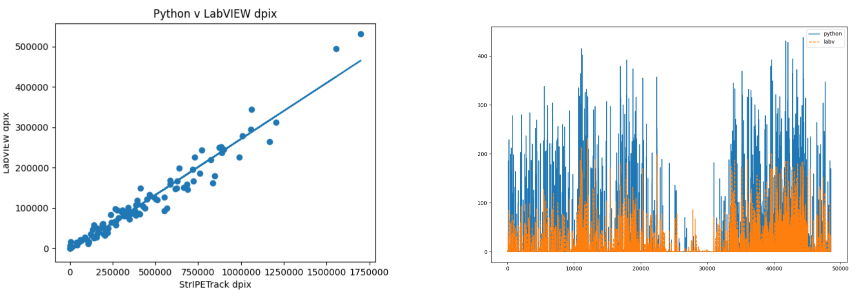

(a) (b)

Fig. S1. (a) Scatterplot where each point represents a fish, and each axis represents sum of delta pixels measured from LabVIEW and StrIPETrack, from one 30 minute movie tracked with both LabVIEW and StrIPETrack. Line represents the line of best fit (Pearson's Correlation: r=0.973, $p < 0.0001$; ICC(3,1) 0.513, $p < 0.0001$, n=95). (b) Single fish response from (a) tracked with both Python (blue) and LabVIEW (orange).

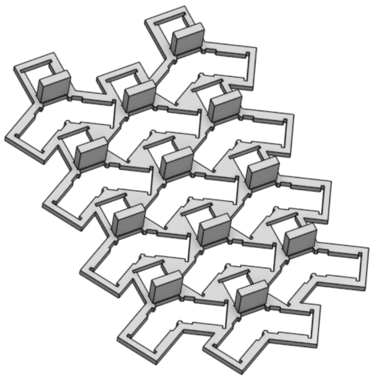

Fig. S2. Example y-maze comb for medium Y size. Zebrafish are habituated in blocked arm, and after 10 minutes the comb is taken out and zebrafish are released.

Biology Open • Supplementary information

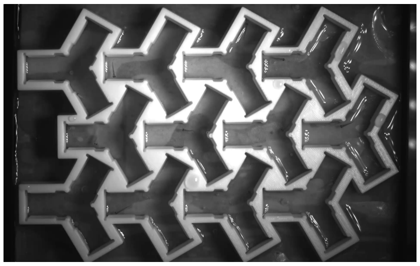

(a)

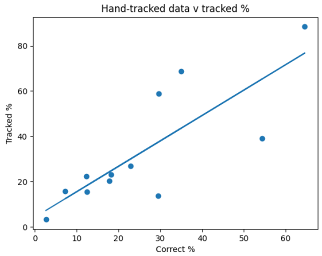

(b)

Fig. S3. Example low contrast image from tracked low-contrast video. When compared with hand-tracked data alternation tetragram %s were highly correlated (r=0.807, p=0.0014).

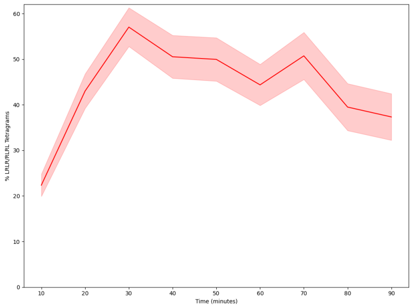

(a)

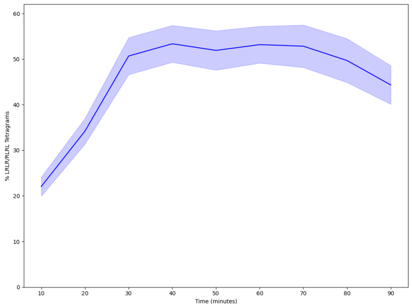

(b)

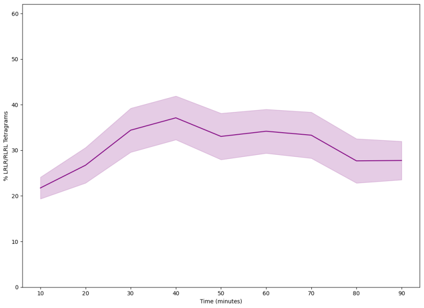

(c)

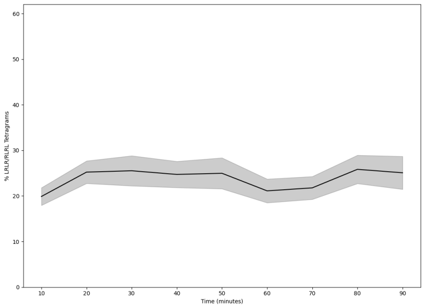

(d)

Fig. S4. Variation in 21 dpf wild type zebrafish from different lines. Alternation tetragram percentage in 4 different lines of wild type zebrafish over 90 minutes of tracking. The mean alternation percentage for each 10 minute bin with the SEM is represented. (a)/red n=38. (b)/blue n=61, (c)/purple n=39, (d)/black n=46

Biology Open • Supplementary information

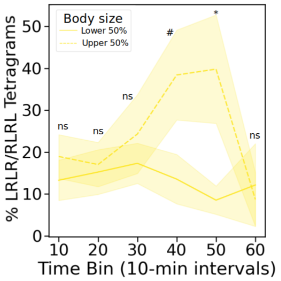

(a)

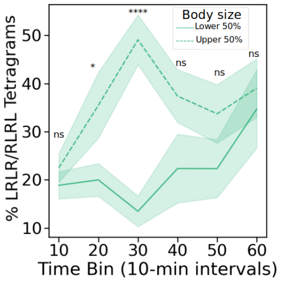

(b)

Fig. S5. Body size influences performance in FMP Y-mazes. Comparison of alternation tetragrams percent of the largest 50% zebrafish (n=12) compared to the smallest 50% zebrafish of 10 dpf zebrafish (n=11) (A) and of the largest 50% zebrafish (n=18) compared to the smallest 50% zebrafish of 21 dpf zebrafish (n=18) (B). The results of two-way ANOVA are denoted above each time bin (ns = not significant, * <0.05, * *<0.01. ***<0.001, ****<0.0001)

Table S1. Benchmarked performance for Intel Core i7, 8GB RAM. naive SSIM is a drop-in replacement for scikit-image SSIM. previous image SSIM and mode image SSIM are described in detail in Supplementary S7.
*Author implementation.

scikit-image SSIM	naive SSIM*	previous image SSIM*	mode image SSIM*
0.034s	0.012s	0.007s	0.005s

Table S2. Accuracy measured by calculating tetragram % for all eight tetragram groups in Y-maze, for 21 dpf zebrafish, n =9. Correlated only between percentage values; does not consider correct number or placement of tetragrams, or correct arm. Each detected centroid from tracking was matched to a specific cell before calculating tetragram percent.

	Bonsai	Stytra	StriPETrack
accuracy	r=0.708, p=3.301e-12	r=0.642, p=1.173e-09	r=0.966, p=4.804e-43
average difference from correct number of detected turns	31286.11	159.55	0.44

Biology Open • Supplementary information
